# Incidental finding of situs ambiguus in a middle‐aged woman

**DOI:** 10.1002/rcr2.1018

**Published:** 2022-08-17

**Authors:** Yung‐Chia Huang, Shih‐Wei Lee, Shih‐Hsin Wu, Mei‐Yin Chen

**Affiliations:** ^1^ Department of Pulmonary Medicine, Taoyuan General Hospital Ministry of Health and Welfare Taoyuan Taiwan; ^2^ Graduate Institute of Biomedical Sciences China Medical University Taichung Taiwan; ^3^ Center of Allergy, Immunology, and Microbiome (AIM) China Medical University Children's Hospital Taichung Taiwan

**Keywords:** chest radiography, congenital abnormality, situs ambiguus, sub‐diaphragmic free air

## Abstract

Situs ambiguus is a rare congenital abnormality with outcomes ranging from asymptomatic to fatal. Here we present a woman with an incidental finding of situs ambiguus hinted by her chest radiograph. This case highlights the importance of actively seeking diagnosis when right sub‐diaphragmic air is noted when viewing a chest radiograph.

## Graphical abstract

We present a case of incidental finding of situs ambiguus hinted by chest radiograph. This case highlights the importance of actively seeking diagnosis when right sub‐diaphragmic air is noted when viewing a chest radiograph.

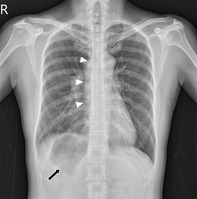

## CLINICAL IMAGE

**FIGURE 1:**
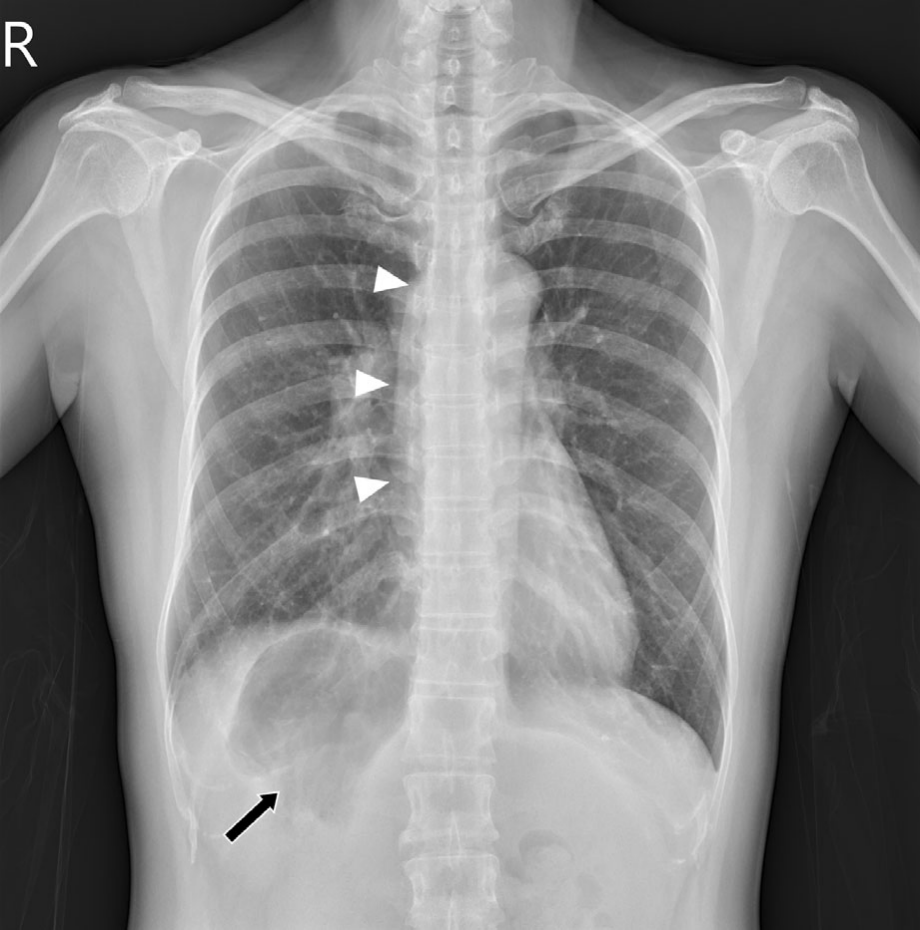
Poster‐anterior view of chest radiography showed abnormal gas collection below the right hemidiaphragm (arrow) and prominent azygous vein (arrowheads).

**FIGURE 2:**
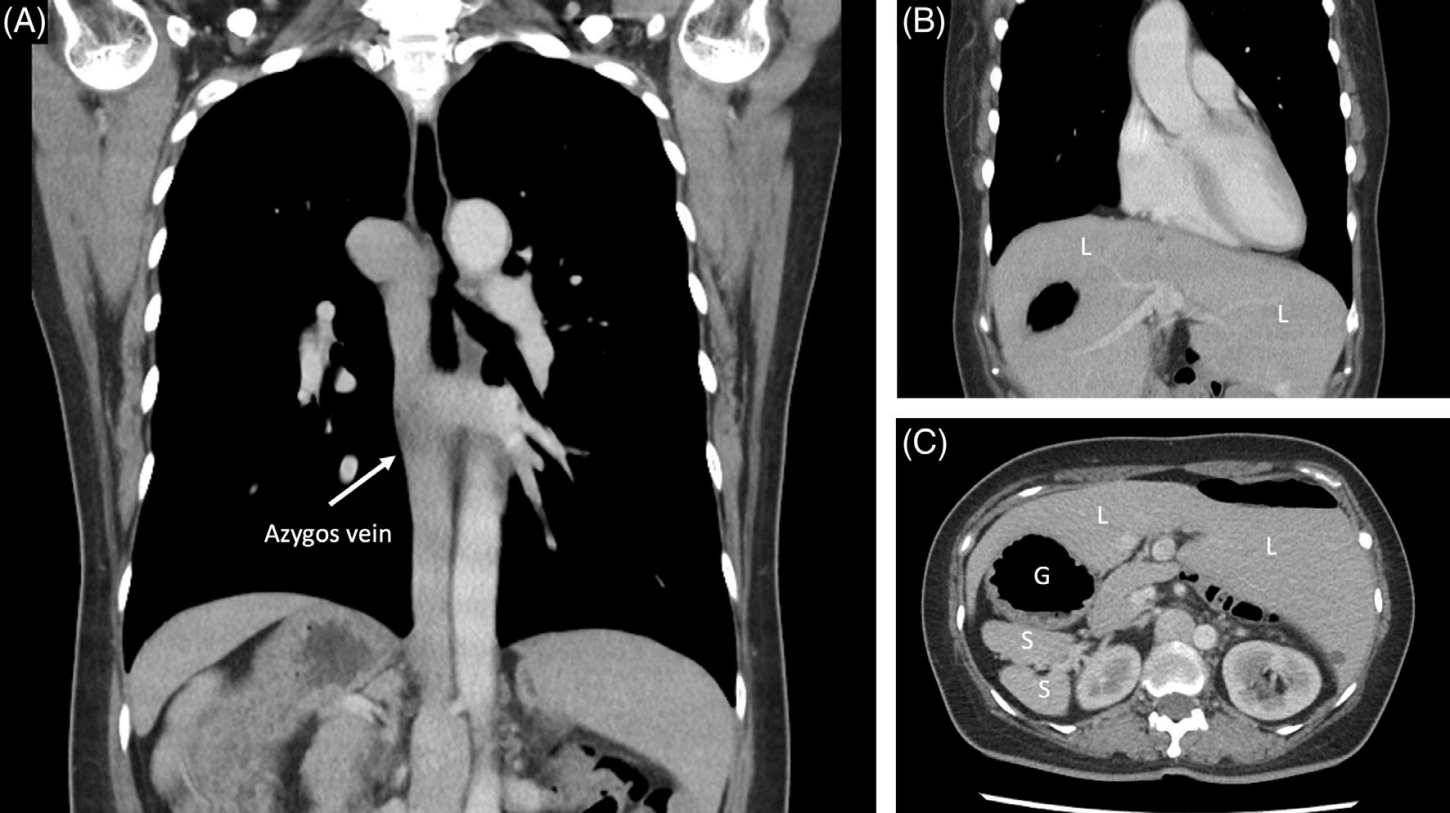
The sagittal view of the contrasted computer tomography revealed azygos continuation of left‐sided inferior vena cava into persistent left superior vena (A) and the liver spanning over the entire diaphragm with missing gall bladder. (B) The horizontal view demonstrated multiple spleens behind the right‐sided stomach. (C) L, liver; G, stomach; S, spleen

An otherwise healthy 46‐year‐old woman presented to the Pulmonary Outpatient Department due to dry coughs for 1 week. On physical examination, she was alert and oriented with stable vital signs. Antitussive medication was given at the office, and a chest radiograph was taken. Her condition was resolved at her follow‐up visit. However, the chest radiograph at the first visit revealed abnormal gas collection below the right hemidiaphragm and a prominent azygous vein which prompted further investigation (Figure 1). A contrasted computer tomography scan showed multiple organ disarrangements, including her liver, stomach, spleen and gallbladder. Azygos continuation of left‐sided inferior vena cava into persistent left superior vena cava is also seen. (Figure 2A–C) The finding is consistent with situs ambiguus.^1^ The collection of gas below the right hemidiaphragm is easily overlooked during the radiography viewing process.^2^ Special attention should be given to the sub‐diaphragmic area and consider further workup if an abnormality is found.

## AUTHOR CONTRIBUTION

Yung‐Chia Huang wrote a manuscript for this manuscript and conducted a literature review. Shih‐Wei Lee contributed to the collection of case information and the revision of the manuscript. Shih‐Sin Wu and Mei‐Yin Chen reviewed the final manuscript. All authors have read and approved the final manuscript.

## CONFLICT OF INTEREST

None declared.

## ETHICS STATEMENT

The authors declare that appropriate written informed consent was obtained for the publication of this manuscript and accompanying images.

## Data Availability

The data that support the findings of this study are available from the corresponding author upon reasonable request.
